# Reduced beta bursting underpins loss of corticomuscular coherence in amyotrophic lateral sclerosis

**DOI:** 10.1093/braincomms/fcaf339

**Published:** 2025-09-09

**Authors:** Katie Yoganathan, Michael Trubshaw, Oliver Kohl, Chetan Gohil, Irene Echeverria-Altuna, Thanuja Dharmadasa, Alicia Northall, Nahid Zokaei, David Lester, Gayle Garcia, Alexis Collins, Benazir Amein, Anna C Nobre, Kevin Talbot, Alexander G Thompson, Mark Woolrich, Martin R Turner

**Affiliations:** Nuffield Department of Clinical Neurosciences, University of Oxford, Oxford OX3 9DU, UK; Oxford Centre for Human Brain Activity, Wellcome Centre for Integrative Neuroimaging, Department of Psychiatry, University of Oxford, Oxford OX3 7JX, UK; Nuffield Department of Clinical Neurosciences, University of Oxford, Oxford OX3 9DU, UK; Oxford Centre for Human Brain Activity, Wellcome Centre for Integrative Neuroimaging, Department of Psychiatry, University of Oxford, Oxford OX3 7JX, UK; Oxford Centre for Human Brain Activity, Wellcome Centre for Integrative Neuroimaging, Department of Psychiatry, University of Oxford, Oxford OX3 7JX, UK; Oxford Centre for Human Brain Activity, Wellcome Centre for Integrative Neuroimaging, Department of Psychiatry, University of Oxford, Oxford OX3 7JX, UK; Oxford Centre for Human Brain Activity, Wellcome Centre for Integrative Neuroimaging, Department of Psychiatry, University of Oxford, Oxford OX3 7JX, UK; Department of Experimental Psychology, University of Oxford, Oxford OX3 7JX, UK; Nuffield Department of Clinical Neurosciences, University of Oxford, Oxford OX3 9DU, UK; Oxford Centre for Human Brain Activity, Wellcome Centre for Integrative Neuroimaging, Department of Psychiatry, University of Oxford, Oxford OX3 7JX, UK; Oxford Centre for Human Brain Activity, Wellcome Centre for Integrative Neuroimaging, Department of Psychiatry, University of Oxford, Oxford OX3 7JX, UK; Nuffield Department of Clinical Neurosciences, University of Oxford, Oxford OX3 9DU, UK; Nuffield Department of Clinical Neurosciences, University of Oxford, Oxford OX3 9DU, UK; Nuffield Department of Clinical Neurosciences, University of Oxford, Oxford OX3 9DU, UK; Nuffield Department of Clinical Neurosciences, University of Oxford, Oxford OX3 9DU, UK; Oxford Centre for Human Brain Activity, Wellcome Centre for Integrative Neuroimaging, Department of Psychiatry, University of Oxford, Oxford OX3 7JX, UK; Department of Experimental Psychology, University of Oxford, Oxford OX3 7JX, UK; Nuffield Department of Clinical Neurosciences, University of Oxford, Oxford OX3 9DU, UK; Nuffield Department of Clinical Neurosciences, University of Oxford, Oxford OX3 9DU, UK; Oxford Centre for Human Brain Activity, Wellcome Centre for Integrative Neuroimaging, Department of Psychiatry, University of Oxford, Oxford OX3 7JX, UK; Nuffield Department of Clinical Neurosciences, University of Oxford, Oxford OX3 9DU, UK; Oxford Centre for Human Brain Activity, Wellcome Centre for Integrative Neuroimaging, Department of Psychiatry, University of Oxford, Oxford OX3 7JX, UK

**Keywords:** magnetoencephalography, MEG, coherence, beta oscillations, beta bursts

## Abstract

Biomarkers of disease activity that holistically capture motor system dysfunction are needed to accelerate drug discovery in amyotrophic lateral sclerosis. Magnetoencephalography is a sensitive, non-invasive measure of cortical neurophysiology. Corticomuscular coherence reflects the functional coupling of cortical oscillations with downstream muscle activity recorded by electromyography. Cortical beta frequency bursting is known to represent a core feature of the neurophysiology underpinning movement. This study aimed to characterize disruption of beta frequency activity in both cortex and muscle to refine the understanding of corticomuscular coherence loss in amyotrophic lateral sclerosis. The study analysed 42 people living with amyotrophic lateral sclerosis and 33 healthy age-matched controls. Participants undertook an isometric hand gripping task during magnetoencephalography. Muscle contraction was measured using bipolar surface electromyography recordings at both forearms. All participants performed 120 trials of the gripper task bilaterally, and 60 trials unilaterally on each side. For each trial type, the mean corticomuscular coherence over trials was calculated for each participant and the groups were compared via cluster-based permutations tests. Beta burst metrics were calculated for the motor cortex (magnetoencephalography) and flexor forearm muscles (surface electromyography) including burst fractional occupancy, burst duration and amplitude. During muscular contraction, beta frequency corticomuscular coherence from the motor cortices contralateral to the gripper task was markedly reduced in amyotrophic lateral sclerosis patients, despite no significant difference in grip strength compared with controls. Source localization analysis showed globally reduced corticomuscular coherence in amyotrophic lateral sclerosis with significant differences in the motor regions contralateral to the engaged hand. There were no significant beta frequency activity changes in the engaged-hand electromyography signal in amyotrophic lateral sclerosis compared with controls. In contrast, analysis of the cortical motor regions revealed reduced rate of beta bursting and higher amplitude during the contraction phase of the task in amyotrophic lateral sclerosis. The corticomuscular coherence disruption in amyotrophic lateral sclerosis appears driven more by cerebral pathology than by muscle denervation. Equal grip strength during the task implies compensatory pathways in disease that are not captured by corticomuscular coherence. Interneuronal dysfunction may underlie the disruption to motor cortex beta bursting. Motor cortical beta frequency metrics have potential as secondary outcome measures in therapeutic trials and need exploration as prodromal markers in asymptomatic individuals genetically predisposed to amyotrophic lateral sclerosis.

## Teaser

Yoganathan *et al*. found reduced connectivity between brain and muscle (corticomuscular coherence) in motor neuron disease patients using magnetoencephalography scanning. They discovered that brain beta burst dysfunction is largely responsible for the changes. Corticomuscular coherence and beta bursts may be useful as an early disease biomarker in treatment trials.

## Graphical Abstract

**Graphical Abstract:**
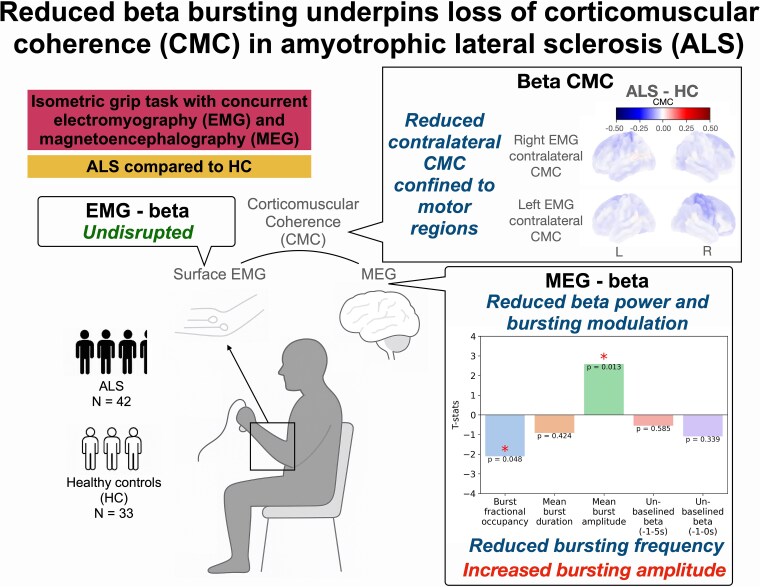

## Introduction

Amyotrophic lateral sclerosis (ALS) is a neurodegenerative disorder characterized by progressive neuromuscular weakness that reflects degeneration of a broader motor-neuronal system spanning cortex to muscle.^1^ A lack of disease modifying therapies is due, in part, to a paucity of biomarkers that reflect disease activity within a pathologically and clinically heterogeneous disorder.

Magnetoencephalography (MEG) offers a non-invasive means of recording cortical neurophysiology with millisecond temporal precision, enabling detailed observation of neural dynamics.^2^ Neurophysiological activity measured by MEG is oscillatory in nature and divided into canonical frequency bands. Cortical beta-band (13–30 Hz) activity is known to be instrumental in the planning, initiation, and maintenance of movement.^3,4^

Cortical beta oscillations exhibit complex spatial dynamics, including wave-like propagation across motor regions during motor tasks like squeezing a gripper.^5^ In the healthy brain, the initiation of movement corresponds with a fall in beta power (beta desynchronization, ‘releasing of the beta brake’), which then rebounds after the movement (beta resynchronization, ‘application of the beta brake’).^6,7^ However, in ALS, the normal modulation of beta power in relation to movement is disrupted.^8^ Disrupted cortical beta oscillations may reflect impairment of efficient motor communication and could represent broader neural communication deficits within the motor system in ALS.^9^

More recent studies have suggested that analyzing short-duration bursts of beta power, termed ‘beta bursts’, allows a richer understanding of beta disruption compared with trial-averaged beta power.^3^ For example, composing one beta power value exists any combination of bursting frequency, bursting length, or bursting amplitude. Changes in individual beta bursting metrics like bursting frequency, have been shown to be directly caused by changes to activity of specific neuronal populations, allowing for increased interpretability of results.^10^ Beta bursts have been previously investigated in neurodegenerative conditions such as Parkinson's disease.^11,12^

Beta oscillations are also visible in muscle activity, detectable through non-invasive electromyography (EMG). Coherence analysis provides crucial insights into the temporal organization between brain activity and muscle contraction. Corticomuscular coherence (CMC) specifically measures the synchrony of electrical activity between the motor cortex and muscle activity during movement.^13,14^ In ALS, evidence of reduced CMC reflected by cortical beta frequency MEG changes has been identified; however, beta bursting properties have not been thoroughly investigated.^15,16^

This study aimed to further characterize CMC in ALS by investigating beta power and beta bursts in both cortex and muscle simultaneously, to better understand ALS neuropathophysiology and to assess the biomarker potential of these metrics.

## Materials and methods

### Participants

This was an observational, cross-sectional study utilizing task-based MEG. Individuals living with ALS (*n* = 42) and healthy age-matched controls [healthy control (HC), *n* = 33] were recruited. HCs were established through prior study cohorts, and generally spouses of patients, or recruited through Join Dementia Research.^17^ They were matched for age and sex, with no significant medical history. ALS patients were recruited from the Oxford Motor Neurone Disease Care and Research Centre and met the Gold Coast criteria for ALS.^18^ Exclusion criteria included a first degree relative with ALS or frontotemporal dementia, or the prior detection of common pathological genetic variants through routine clinical screening. All participants underwent cognitive screening using the Edinburgh Cognitive and Behavioural ALS screen (ECAS). Clinical assessments included the revised ALS Functional Rating Scale (ALSFRS-R, 0–48), where a lower score indicates greater disability, and an upper motor neuron (UMN) score derived from the number of pathological reflexes detected on examination (0–15).^19^ Handedness was assessed using the Edinburgh Handedness Inventory.^20^ Written informed consent was obtained from all participants, and the study received approval from the National Research Ethics Service Committee (17/SC/0277).

### MEG acquisition

MEG data were acquired using an Elekta Neuromag system with 306 channels, sampling at 1000 Hz. A Polhemus EastTrach 3D tracking system was used to record the shape of each participant's head relative to fiducial points on the nasion and right and left preauricular areas before starting the MEG scan. Continuous tracking of head position during the scan was achieved with five head position indicator (HPI) coils, aligned with the nasion and bilateral supra-orbital and posterior auricular landmarks. The positions of the HPI coils and fiducials were digitized to create a subject-specific cartesian head coordinate system. Cardiac activity, eye movements, and eye blinks were monitored using ECG electrodes on the right clavicle and left hip bone, and electrodes near the eyes for recording horizontal and vertical electro-oculograms.

### MEG gripper task and procedure

Participants engaged in three isometric gripper tasks (bilateral, unilateral—right hand grip, unilateral—left hand grip), accompanied by concurrent MEG scanning and bipolar EMG recordings at both forearms, with electrodes placed over the flexor digitorum superficialis muscles.^15^ For bilateral gripper tasks, visual cues included two bars on either side of a central fixation cross, indicating the matching force to be exerted by each hand, represented by the height of red lines on each bar. All participants were required to maintain a light grip force of between 12 and 17 Newtons for 3 s after the trigger was shown. Low force levels were utilized because prior research has demonstrated that motor cortical neurons exhibit the highest sensitivity within this force range.^21,22^ The grip strength was recorded using MEG-compatible fibre-optic force sensors. For unilateral tasks, a single bar was presented corresponding to the active hand. Participants completed 120 bilateral trials (60 per force condition) and 60 unilateral trials per side (30 per force condition for each side), with a 2 s rest interval between trials. Direct visual feedback was provided throughout the task and graphical representation can be found in Supplementary Fig. 1.

### MRI acquisition

Participants underwent a T1-weighted structural MRI scan with a Siemens Trio 3T (settings: three-dimensional, whole-brain, magnetization-prepared rapid-acquisition gradient echo sequence, repetition time = 2040 ms, echo time = 4.7 ms, flip angle 8°, 1 mm isotropic resolution, 6 min acquisition time) on the same day for MEG co-registration.

### Data preprocessing

For data processing and analysis, the Oxford Software Library (OSL) version 0.6.0 pipeline, which is built on MNE Python^23^ was used.^24^ Initial MEG data preprocessing was conducted using MaxFilter software version 2.2, which included temporal signal space separation, head movement compensation, and automatic detection of bad channels to enhance data quality by minimizing noise and artefacts.^25^ The EMG signals and the Maxfiltered MEG were then bandpass filtered between 0.5 and 125 Hz using a fifth-order IIR Butterworth filter to retain the frequency band of interest. Additionally, specific line noise frequencies at 50 and 100 Hz were attenuated through notch filtering. To streamline further processing, the EMG and MEG sampling rate was reduced to 250 Hz. Bad data segments in the EMG, magnetometers, and gradiometers, based on their deviation from expected conditions specific to each sensor-type, were identified and excluded. MEG channels demonstrating consistently poor performance were also marked as bad using the same significance criterion.

After data cleaning, co-registration was performed using OSL's RHINO tool to align each subject's structural MRI data with their Polhemus head-shape points (>100) and fiducials (left preauricular, right preauricular, nasion). Forward modelling was then carried out using the boundary element method to construct a single-layer model of the inner skull surface. A bandpass filter between 1 and 80 Hz was applied to the MEG data prior to source localization. Coregistered and cleaned MEG data were then beamformed to a regular, volumetric 8 × 8 × 8 mm dipole grid using a linearly constrained minimum variance beamformer with a data covariance matrix regularized to a rank of 60, with dipole orientations calculated by maximizing each dipole's power. Next, the predefined Glasser52 atlas in MNI space was used to compute 52 parcel time courses.^26^ Principal component analysis was applied to the time series from all voxels assigned to a parcel, and the first principal component was used for the parcel time course. Spatial leakage was reduced by applying symmetric orthogonalization.^27^

For each participant, task-specific MEG data files were segmented into epochs based on detected event triggers. Specific channels were selected for analysis, which included parcellated brain regions and auxiliary channels such as EMG and gripper channels. The epochs were defined from −1 to +5 s relative to the event onset producing a 6 s time course for each trial. Epochs exhibiting artefacts or excessive noise, identified through variability metrics, were excluded from the analysis.

### Behavioural analysis

Behavioural metrics extracted from the gripper data included reaction time (time from trigger to gripper squeeze), proportion of correct responses (the proportion of trials that achieved the requested grip strength indicated by the height of the horizontal lines on screen), grip length (the total duration of grip), and grip strength (the mean grip strength over and above the required grip strength during the grip-on period).

### CMC estimation

CMC is a classical coherence measure (i.e. a time-averaged value) and returns a matrix of coherence values between regions of interest. CMC can be calculated across a frequency range (e.g. beta) as in the ‘Whole-brain beta CMC topography’ section or for individual frequencies at regular intervals (e.g. 1.25 Hz intervals) as in the ‘Motor CMC’ section.

### Motor CMC

For each task (bilateral, unilateral right, unilateral left) and participant, epoched EMG and MEG data were extracted. Data from motor cortices (index 4 and 30 of the Glasser52 parcellation, corresponding to the right and left superior somatosensory and motor cortex, respectively) and EMG channels (right and left) were baseline-corrected separately (the trial average was subtracted from each time point using the equation *X*(*t*) = *U*(*t*)−*M*(*t*) where *U*(*t*) is the original (trial-averaged) signal time series and *M*(*t*) is the average across trials at each time point (*t*)). This method of baseline correction was employed to allow for examination of the modulation of CMC in response to the task, rather than raw (un-baselined) CMC, which would be confounded by the baseline CMC value. EMG signals were rectified. CMC was estimated between 8 and 40 Hz at 1.25-Hz intervals across the 2- to 4-s period post-stimulus using the multitaper method to build a CMC matrix (window length = 200 samples). Right and left contralateral and ipsilateral CMC were calculated by taking the corresponding edges of the matrix.

### Whole-brain beta CMC topography

A further analysis estimated *beta* CMC, pairwise, between each EMG channel and each brain region thereby producing a topographical map of CMC strength over the cortical surface. CMC was calculated as detailed above, but this time *mean beta* CMC was calculated by taking the average CMC across 13–30 Hz and 1–3 s post-trigger (2–4 s of each trial) to create a whole-brain CMC connectivity matrix.

### Trial-averaged beta power and beta burst analysis

Trial-averaged beta power and beta bursts were estimated for each of the three tasks (bilateral, left grip, right grip). To estimate power spectral density, time courses from the bilateral motor cortices and EMG channels were first standardized by converting them to *z*-scores (i.e. subtracting the mean and dividing by the standard deviation). The MNE-Python package was used to estimate a time–frequency representation (Morlet wavelet) of the standardized (*z*-transformed) and baseline-corrected (per-trial average subtracted) raw MEG and EMG time courses. Power in the beta (13–30 Hz) frequency band was calculated from the time–frequency transform of each epoch. The average beta power across trials was calculated to produce a per-participant mean beta power time course.^12^

To investigate whether baseline beta power was different between the groups, mean standardized, but un-baseline-corrected beta power was calculated between −1 and 5 s and −1 and 0 s, of each trial across right and left superior somatosensory and motor regions (the same period was used for CMC estimation).^28^

Burst detection involved filtering the raw data within the beta frequency range (13–30 Hz), extracting amplitude envelopes via Hilbert transformation, and normalizing these envelopes using the per-trial-mean. Bursts were defined by applying a 75th percentile threshold and excluding events shorter than the minimum duration of one cycle of the lowest frequency. For each participant, burst fractional occupancy [the proportion of time (between 1 and 3 s post trigger) taken up by beta bursts], mean burst duration (the average length of each burst), and mean burst amplitude (the average power of a burst when it occurred) were computed.

### Statistical analysis

Python, GLM Tools, MNE, and Scipy packages were used for all statistical analyses.^29-33^ Non-parametric permutation testing, using 5000 permutations, was chosen to test for significant differences in neurophysiological data between HC and ALS as this method makes very limited assumptions on the distribution of the data.^34^

MNE's cluster-based permutation testing was employed for the motor CMC, beta power time course and beta burst time course analyses.^35-37^ Clusters were defined in the two dimensions of time and frequency. Multiple comparisons were accounted for across frequencies (for the motor CMC analysis) and across timepoints (for the beta power and beta burst time course analyses). Additionally, Bonferroni correction was applied to account for multiple comparisons across tasks (bilateral, unilateral right, unilateral left) and hemispheres (right motor CMC and left motor CMC). Cluster *P*-values of <0.05 were reported after all multiple comparison corrections.

Permutation-based general linear models (GLMs) were constructed for the whole-brain beta CMC topography analysis and to compare beta power and beta burst metrics. Multiple comparisons were accounted for using the maximum t-statistic correction method across tasks, hemispheres and regions (for the topography analysis) and across metrics for the beta power/bursts analysis.^34^ Age, sex, and missing structural MRI were included as (confound) regressors in the model. Contrast of Parameter Estimates (copes) was estimated to illustrate the size of effect of each contrast.^34^ A design matrix can be found in Supplementary Fig. 2. Results were reported using the notation [*t*-statistic(degrees of freedom), *P*] when *P* < 0.05 after multiple comparison corrections.

Demographics, clinical scores (e.g. ECAS scores), and behavioural data (e.g. grip reaction times) were compared between groups using Welch's *t*-test (for continuous) and the Chi-squared test (for categorical) with Bonferroni correction across measures. CMC correlation with clinical scores was estimated using Pearson correlation and the Pearson correlation coefficient reported using the notation *r*.

## Results

All *P*-values are reported after multiple comparisons correction.

### Demographics

There was no significant group difference in age, handedness, education, or maximum voluntary contraction grip strength (*P* > 0.1) (Table 1). Further characteristics characterizing the ALS cohort can be found in Supplementary Table 1.

**Table 1:** Cohort characteristics

Mean ± SD	ALS (*n* = 42)	Healthy controls (*n* = 33)	Adjusted *P*-value
Age (years)	62 ± 12	62 ± 16	1.00
Males (%)	65	49	0.19
Right MVC strength (*N*)	25 ± 15	31 ± 11	0.33
ECAS (/136)	113 ± 9	118 ± 10	0.11
ALS-specific (/100)	85 ± 6	90 ± 8	0.14
Non-ALS specific (/36)	27 ± 5	29 ± 4	1.00
Full time education age (years)	20 ± 5	23 ± 5	0.28

This table summarizes the demographic and clinical characteristics of the study participants, comparing amyotrophic lateral sclerosis (ALS, *N* = 42) with healthy controls (*N* = 33). There were no statistically significant differences in age, sex distribution, handedness, education, MVC strength, or cognitive scores between the patient and healthy control groups, as indicated by the adjusted *P*-values via Bonferroni correction.

MVC, maximum voluntary contraction; ECAS, Edinburgh Cognitive and Behavioural ALS screen; N, Newtons.

### Behavioural analysis

#### There were no significant differences between ALS and HC in reaction time, proportion of correct responses, or grip strength

**Figure 1:**
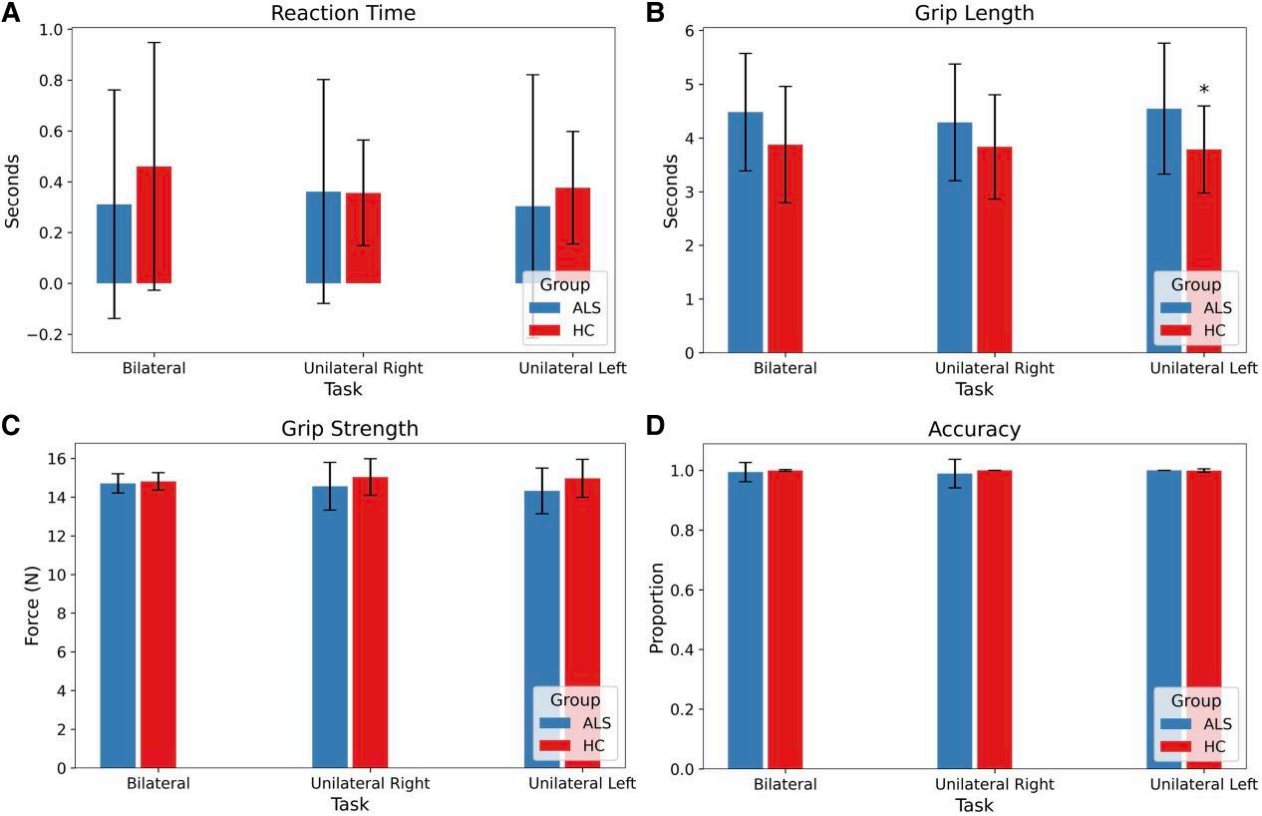
**Behavioural analysis**. Comparison of behavioural metrics: reaction time (**A**), grip length (**B**), grip strength (**C**), and accuracy (**D**) across each task comparing ALS (*N* = 42) and HC (*N* = 33) groups. The analysis was conducted using *t*-tests, with *P*-values adjusted for multiple comparisons using the Bonferroni correction across three tasks (bilateral, right, and left) and four metrics (reaction time, grip length, grip strength, and accuracy). An asterisk (*) indicates a significant difference with *P* < 0.05. A significant difference was observed in the ALS group, with increased grip length in the unilateral left task (*P = 0.017*) compared with HCs.

Median reaction time revealed no significant differences between the groups in bilateral and unilateral tasks (Fig. 1A). There was no significant difference in the proportion of correct responses in bilateral and unilateral tasks, showing that both groups achieved the same minimum grip strength (Fig. 1D). There was no significant difference in grip strength (over and above minimum required grip strength) between the groups in bilateral and unilateral tasks (Fig. 1C). The ALS group did, however, show increased grip length in the unilateral left task (*t* = 3.307, *P* = 0.017), but not in bilateral (*t* = 2.416, *P* = 0.221) or unilateral right (*t* = 1.928, *P* = 0.694) tasks (Fig. 1B).

### CMC analysis

#### Motor CMC

##### CMC in contralateral motor cortex was reduced in ALS compared with controls

**Figure 2:**
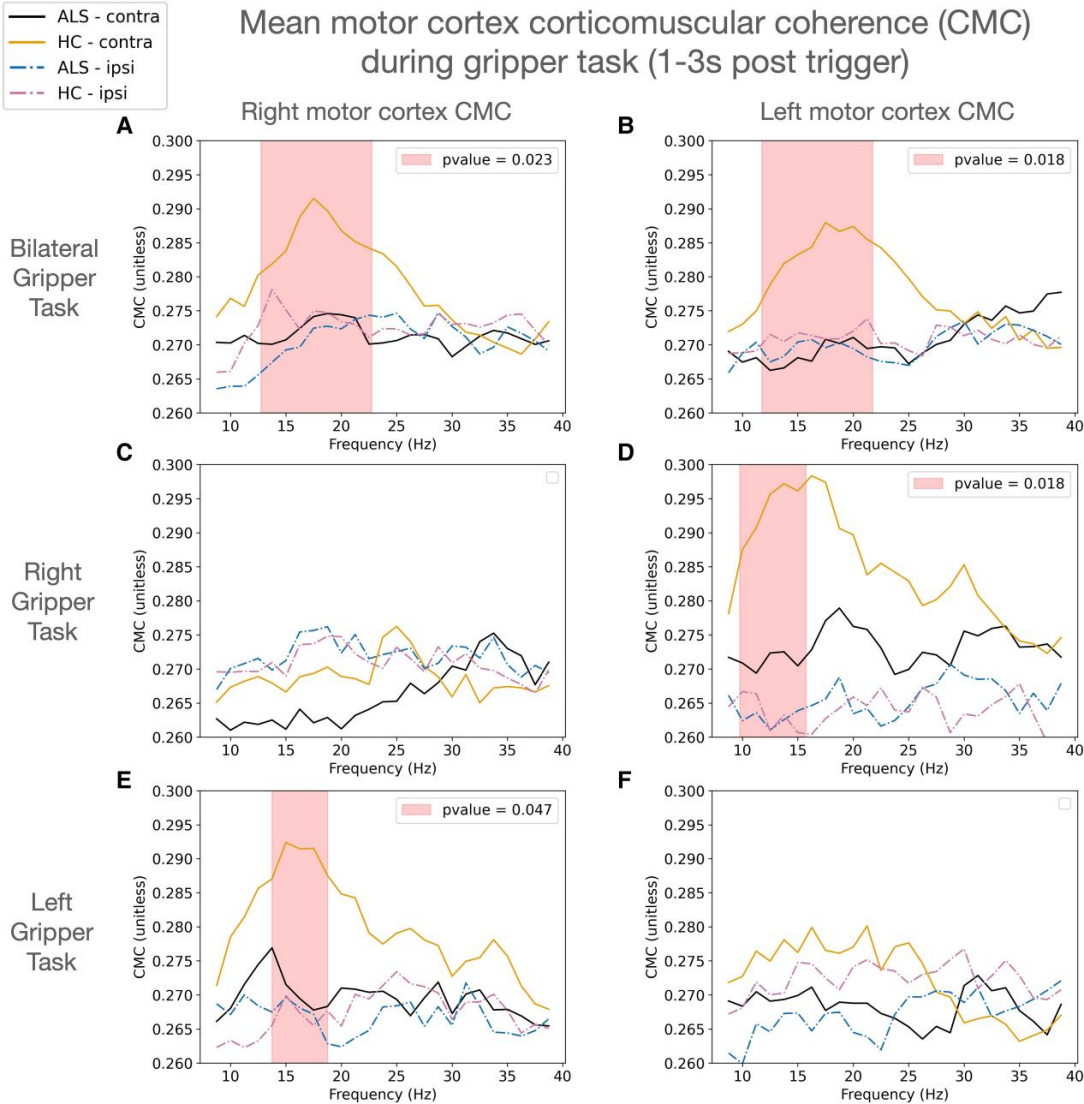
**Motor cortex corticomuscular coherence (CMC)**. CMC in the motor cortices during the gripper task, averaged across the contraction phase of each epoch (1–3 s post trigger). The plots show CMC for both contralateral (contra) and ipsilateral (ipsi) hemispheres across bilateral (**A** and **B**), right (**C** and **D**), and left (**E** and **F**) gripper tasks. Shaded areas indicate the frequency bands where significant differences between ALS (*N* = 42) and HC (*N* = 33) from cluster-permutations in contralateral CMC were found, with corresponding adjusted *P*-values highlighted. Contralateral CMC was reduced in both the right (13–22.5 Hz, *P = 0.023*) and left (12–21.5 Hz, *P = 0.018*) motor cortices during the bilateral gripper task. For the right gripper task, contralateral CMC was reduced in ALS in the left motor cortex (9.9–15.5 Hz, *P = 0.018*). For the left gripper task, contralateral CMC was reduced in the ALS group in the right motor cortex (14–19 Hz, *P = 0.047*) compared with HC.

During the bilateral gripper task, contralateral CMC was diminished in both the right (13–22.5 Hz, *P = 0.023*) (Fig. 2A) and left (12–21.5 Hz, *P = 0.018*) (Fig. 2B) motor cortices in individuals with ALS compared with HC. In the right gripper task, individuals with ALS showed significantly reduced CMC compared with HC in only the left motor cortex (9.9–15.5 Hz, *P = 0.018*) (Fig. 2D); and for the left gripper task, individuals with ALS showed significantly reduced CMC compared with HC in only the right motor cortex (14–19 Hz, *P = 0.047*) (Fig. 2E).

#### Whole-brain beta CMC topography

##### Significantly reduced CMC in ALS compared with controls was limited to motor cortical areas

**Figure 3:**
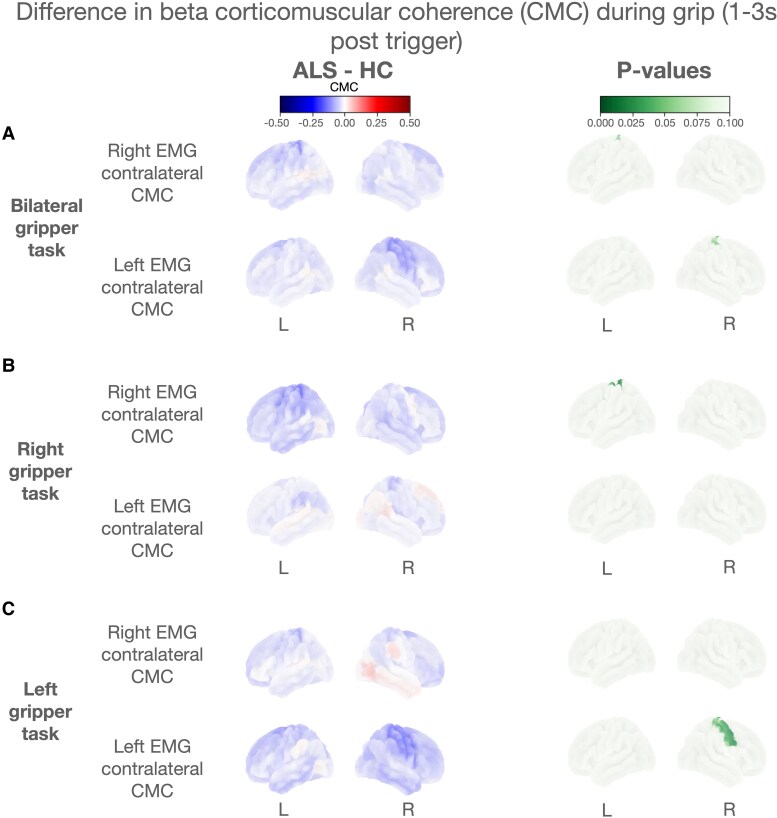
**Difference in beta corticomuscular coherence (CMC) topography during gripper task**. Topographical maps illustrating the difference in contralateral beta CMC between ALS (*N* = 42) and HC (*N* = 33) groups during gripper tasks averaged across the contraction phase of each epoch (1–3 s post trigger). The first column shows the difference in CMC between the groups. The second column shows adjusted *P*-value maps from the permutations-based GLM comparing CMC between the two groups. *P*-values < 0.1 are shaded in green. The maps show coherence in the right and left motor cortices for bilateral (**A**), right (**B**), and left (**C**) gripper tasks. Contralateral CMC is displayed for both right and left EMG for each task. CMC values are color-coded, with a scale from blue to red indicating a negative or positive difference between ALS and HC. The ALS group showed reduced CMC globally, but significant differences between HC (*P* < 0.05) were only present in the motor regions contralateral to grip side.

Whole-brain beta CMC analysis revealed that reduced CMC in individuals with ALS compared with HC was limited to contralateral motor regions in all tasks (Fig. 3). For the bilateral task, these contralateral CMC reductions were limited to the left and right superior somatosensory cortices [non-significant (*t*(78) = −0.831, *P* = 0.083) and (*t*(78) = −0.815, *P* = 0.090), respectively] (Fig. 3A). For the right gripper task, left superior somatosensory cortex contralateral CMC showed significant reductions in ALS compared with HC [*t*(78) = −0.997, *P* = 0.020] (Fig. 3B). For the left gripper task, ALS showed significant reduction in ALS compared with HC in right premotor area contralateral CMC [*t*(78) = −0.915, *P* = 0.042] and in superior and inferior right sensorimotor contralateral CMC (non-significant [*t*(78) = −0.850, *P* = 0.072] and [*t*(78) = −0.871, *P* = 0.062], respectively) (Fig. 3C). A topography heatmap showed that beta CMC was uniformly reduced in ALS compared with HC in an area confined to motor regions (Supplementary Fig. 3).

#### Trial-averaged beta power

##### Beta power was disrupted in motor cortex but not sEMG

**Figure 4:**
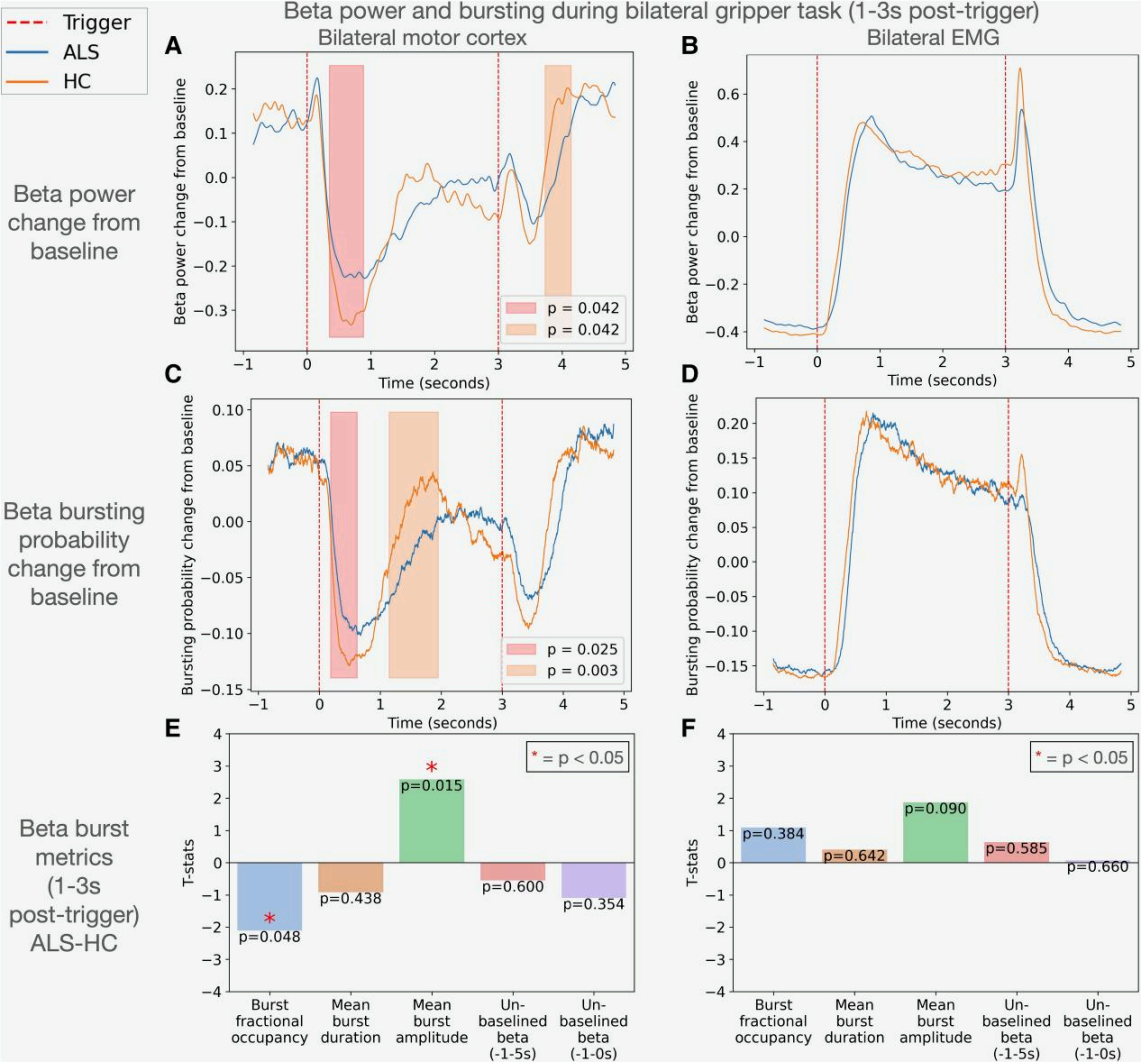
**Beta power and bursts in bilateral task**. Differences in beta metrics between ALS (*N* = 42) and HC (*N* = 33) in motor cortex (MEG, first column) and muscle (EMG, second column). (**A**) illustrates beta power modulation in the motor cortex, with baseline correction across the entire trial period. Time is shown in seconds with the ‘grip’ trigger set at 0 s. Shaded vertical bars indicate periods of significant differences between groups with adjusted *P*-values marked (*P* < 0.05) from cluster-permutations analyses. ALS showed a significantly smaller desynchronization (increased beta power 0.352–0.888 s) compared with HC in response to the ‘grip’ trigger. Beta rebound was significantly reduced (lower beta power) and delayed in ALS in response to the ‘grip off’ trigger (3.736–4.148 s). (**B**) displays beta power modulation in the EMG, also baseline-corrected across the entire trial period. Both groups’ beta power increased in response to the ‘grip’ trigger then decreased after the ‘grip off’ trigger. There were no significant differences in EMG beta power between the two groups over time. (**C** and **D**) compare beta bursting probabilities over time in both groups, with baseline correction as previously. (**C**) displays bursting probabilities in the motor cortex, in which the ALS group, in response to the ‘grip’ trigger, showed significantly increased beta bursting probability (0.184–0.620 s) followed by significantly reduced beta bursting probability (1.144–1.952 s). (**D**) illustrates no significant differences in EMG beta burst probabilities between the two groups. (**E** and **F**) display bar graphs comparing beta burst metrics between ALS and HC. The metrics displayed include burst fractional occupancy, mean burst duration, mean burst amplitude, un-baselined beta power (averaged between −1 and 5 s of the trial) and un-baselined beta power (averaged between −1 and 0 s of the trial). A significant difference (*P* < 0.05) is indicated by an asterisk (*).

In the motor cortices in response to the bilateral ‘grip’ trigger, individuals with ALS exhibited a significantly smaller beta desynchronization, indicated by increased beta power (averaged across left and right motor cortices) between 0.352 and 0.888 s (*P* = 0.042) (Fig. 4A). Furthermore, the beta rebound after the ‘grip off’ trigger was also notably reduced and delayed in ALS between 3.736 and 4.148 s (*P* = 0.042) (Fig. 4A). There were no significant differences in motor cortex beta power during the contraction phase between groups. For EMG, beta power in both groups increased following the ‘grip’ trigger and decreased after the ‘grip off’ trigger (Fig. 4B). There were no statistically significant group differences in EMG beta power fluctuations in response to the gripper task (Fig. 4B**)**.

In the unilateral tasks, a similar pattern of changes to the bilateral task was seen in ALS, but this only reached statistical significance in the right grip task (Supplementary Fig. 4) where both the contra- and ipsilateral motor cortices showed impaired beta power fluctuations. See Supplementary Fig. 5 for left gripper task.

#### Beta bursts

##### Beta bursts were disrupted in motor cortex

In the bilateral task, beta bursting probabilities averaged across both motor cortices were increased in the ALS group compared with HC between 0.184 and 0.620 s (*P* = 0.025), followed by a significant decrease from 1.144 to 1.952 s in response to the ‘grip’ trigger (*P* = 0.003) (Fig. 4C). In contrast, no significant differences were observed in EMG beta burst probabilities between the groups (Fig. 4D).

Similarly to the bilateral task, the unilateral task analysis revealed disruptions in beta bursting probabilities in the right gripper (Supplementary Fig. 4E and F) and left gripper (Supplementary Fig. 5E and F) tasks in both contra- and ipsilateral motor cortices. The right gripper task additionally revealed increased beta bursting in the left (non-gripping) EMG (0.90–0.98 s, *P* = 0.041).

In the bilateral task, comparative analysis of motor cortex beta burst metrics between ALS and HC during the 1–3 s interval post-trigger revealed a significant reduction in burst fractional occupancy in the ALS group compared with HC (*P* = 0.048) (Fig. 4E). Furthermore, there was a significant elevation in mean burst amplitude within the ALS group (*P* = 0.015) (Fig. 4E). While EMG beta burst metrics did not show significant differences overall, an absolute increase in mean beta burst amplitude was noted in the ALS group compared with HC (*P* = 0.090) (Fig. 4F). There were no significant differences in mean un-baselined beta power (−1 to 5 s) or un-baselined beta power (−1 to 0 s) between groups.

The pattern of reduced burst fractional occupancy in contralateral motor cortex in ALS was replicated in the unilateral tasks, but statistically significantly reduced fractional occupancy was seen only in the right gripper task (*P* = 0.048 (Supplementary Fig. 4J**)**) (compared with (*P* = 0.115) in the left gripper task, Supplementary Fig. 5I). Furthermore, in the right gripper task, increases in ALS of un-baselined beta power averaged between −1 and 5 s (*P* = 0.021) and −1 and 0 s (*P* = 0.017) were observed in the left (non-gripping) EMG (Supplementary Fig. 4L). Right (non-gripping) EMG un-baselined beta power in the left gripper task was also increased but did not reach statistical significance (*P* = 0.086 and *P* = 0.560 respectively, Supplementary Fig. 5K).

#### Clinical correlations

##### There were no significant correlations between CMC and clinical scores in the ALS group

Pearson correlation analyses revealed no significant correlation between contralateral beta-band CMC in the bilateral task and several clinical parameters including ALSFRS-R, UMN score, neurofilament light chain (NfL) levels (measured within 1 month of MEG), duration, progression rate, and ECAS scores (Supplementary Fig. 6). Additionally, there were also no significant correlations between beta-band CMC and metrics of right and left grip strength, years of education, ALS-specific and non-ALS-specific ECAS scores, or forced vital capacity (Supplementary Fig. 7). A supplementary analysis correlating CMC to ALSFRS-R handwriting sub-score also returned insignificant (*P* > 0.1).

## Discussion

During muscular contraction, beta frequency CMC from the motor cortices contralateral to the gripper task were markedly reduced in ALS compared with controls, with marked disruptions in the cortical beta power and bursting in contrast to a lack of change in muscle recordings. The latter implies more complex, perhaps extra-motor, compensatory mechanisms associated with the relative preservation of sEMG beta signal, not captured by CMC.

### Previous studies of CMC

Beta cortical signals, shaped by the dendritic architecture of corticospinal neurons, modulate descending motor messages to spinal motor neurons, which result in muscular contraction.^38,39^ Impairments in beta CMC, the coherence between beta oscillations in cortex and muscle, are evident during grip tasks, where ALS patients and even asymptomatic gene carriers show reduced CMC compared with HCs.^15,16^ These reductions underscore a widespread disruption in motor connectivity, which may also extend to intermuscular and inter-hemispheric interactions.^15,40^ Building on these observations,^15^ the present study also included unilateral tasks and showed, through source localization, that CMC reductions are limited to contralateral motor regions in both bilateral and unilateral tasks.

### Laterality

The healthy motor system is inherently asymmetric, with the majority of central nervous system motor fibres supplying the right peripheral nervous system (right motor system), even after controlling for handedness.^41-43^ A recent review summarized the evidence for lateralized ALS pathophysiology.^44^ Some studies have suggested preferential damage to the right motor system.^45-48^ This study supports this contention. Of the unilateral tasks, only the right gripper task revealed significant changes in cortical bursting, perhaps linking to increased right motor system vulnerability in ALS. Furthermore, in the right gripper task, ALS patients showed increased EMG beta bursting in the left (non-gripping) EMG, suggesting a failure of top-down inhibitory control resulting in aberrant muscle activation on the ipsilateral side.

The unilateral tasks analysis showed that cortical beta power and bursting findings were similar across both contralateral and ipsilateral motor cortices, even when only one hand was squeezed. This provides functional evidence for disruption to trans-callosal fibres in ALS.^49^ MRI evidence suggests that functional disturbances of trans-callosal pathways may precede microstructural changes in the corpus callosum.^50^

Previous studies have explored hemispheric contributions to CMC during unilateral hand movements. Chen *et al*.^51^ reported contralateral CMC over primary motor cortex for both hands, and additionally observed CMC over the supplementary motor area during right-hand precision grip tasks, suggesting task-specific recruitment of midline motor areas.^51^ The present study found that ALS CMC reduction was limited to the contralateral motor regions, suggesting that CMC disruptions primarily reflect damage to the corticospinal tract rather than the cross-callosal fibres which support hemispheric mirroring.

### CMC as a biomarker of cortical pathology

CMC in stroke patients is markedly reduced immediately post insult, followed by a steady improvement, which correlates with clinical improvement.^13,52-54^ Transcranial direct current stimulation, a targeted electrical stimulation of the cortex, has been used to modulate beta CMC, and may serve as a physiological marker for tracking or predicting the effects of therapeutic interventions.^55^ In Parkinson's disease, CMC is preserved in early disease and is modulated by symptomatic treatments like L-Dopa and deep brain stimulation (DBS).^13,56^ Early application of CMC monitoring and modulation has been linked with higher therapeutic success.^57-59^ Given that the present study observed no significant correlation between ALS clinical measures and contralateral beta CMC, it may therefore represent a much broader measure of loss of motor system integrity in the context of a neurogenerative disorder such as ALS compared with an acute insult such as stroke.

### A cortically driven process of degeneration

In addition to its potential as a dynamic biomarker, reduced CMC may reflect specific pathophysiological mechanisms within the motor system in ALS. Studies using transcranial magnetic stimulation (TMS) have demonstrated cortical hyperexcitability as an early and defining feature of ALS.^60-63^ Resting-state M/EEG studies similarly report altered oscillatory activity, including increased gamma power and disrupted connectivity.^8,64^ In this context, reduced CMC may arise from both impaired corticomotor neuron output and altered network synchronization, potentially reflecting a breakdown in the coherent integration of cortical and peripheral signals. Importantly, cortical hyperexcitability and cortical motor neuron dysfunction are not mutually exclusive; rather they may represent parallel or sequential processes in ALS pathogenesis. Thus, diminished CMC could index both decreased functional integrity of corticospinal projections and maladaptive cortical dynamics.

Beta bursts, short-transient periods of high-power beta oscillations, are more closely functionally coupled to movement^65^ and more predictive of behaviour^66^ than oscillatory beta power. Research indicates that the altered dynamics of beta bursting seen in Parkinson's disease can improve with therapeutic interventions.^67,68^ Specifically, studies have shown that in untreated Parkinson's disease patients are more likely to experience longer and more intense beta bursts compared with those who have received treatment.^69,70^

During the tonic grip phase of the gripper task, ALS showed no change in overall beta power, but beta bursting probability, burst fractional occupancy and burst amplitude were all disrupted. West *et al*. performed dynamic causal modelling of cortical beta bursts and identified that reduced connectivity between deep pyramidal and inhibitory interneurons, and middle pyramidal and inhibitory interneurons results in increased beta bursting.^10^ It is therefore likely that the reduced fractional occupancy of bursts in ALS reflects changes in the inhibitory influence of local interneuronal circuits. This pathogenic hypothesis (reviewed in Turner and Kiernan^71^) has a growing foundation of evidence, including PET,^72,73^ histopathology^74^ and TMS.^75^ The lack of significant alterations to either raw beta power or any of the burst metrics in the sEMG signals supports a ‘top-down’ source for the peripheral neuromuscular dysfunction in ALS.

Although previous resting-state MEG work has shown that beta power is reduced in ALS,^76^ this study found no difference between ALS and HC in un-baselined beta power across the whole trial (−1 to 5 s) and un-baselined beta power pre-movement (−1 to 0 s). The reason for this negative result may be that beta power is only reduced in the resting state in ALS, whereas averaged across a task, mean beta power is similar to HC levels. Prior to each movement (−1 to 0 s), ALS patients may have reached a higher beta value relative to their true resting-state beta value due to successive trials running sequentially, not allowing beta to fall back to its resting-state baseline. Alternatively, beta power may have been truly reduced during this period, but the measurement was too noisy to detect a significant difference due to the short time period (1 s) from which beta power was calculated.

### Limitations

The lack of correlation between CMC and NfL levels is likely to reflect the distinct physiological domains captured by these two biomarkers. NfL is a non-specific indicator of the rate of global axonal loss across a range of neurological disorders,^77^ while CMC measures the functional synchronization between cortex and muscle, specifically reflecting the integrity of corticospinal and sensorimotor networks during motor performance. CMC did not correlate with behavioural metrics, and it is likely that changes in behaviour are due to disruption of more complex interactions between intra- and inter-regional neurons not quantified by the single measure of CMC. None of our patients had unilateral symptoms. Ideally, a longitudinal study in those with unilateral limb weakness might advance understanding of the bihemispheric spread of pathology. The heterogenicity of this cohort may have contributed to the lack of clinical correlations.

The threshold of 75% was used to detect beta bursts and was chosen based on previous work.^10,70,78^ Objective assessment of lower motor neuron (LMN) function at the time of MEG acquisition was not made, precluding examination of the relationship between CMC and LMN integrity, which is relevant to motor system dysfunction in ALS. Individual structural MRI data were unavailable for eight participants (six ALS and two HCs). For these individuals, source analysis was conducted using the MNI152 standard brain template. The spatial accuracy of this MEG data was likely reduced, but a regressor to the GLM was used to mitigate any systematic bias introduced by the method.

## Conclusion

Changes in CMC may occur prior to overt weakness and could serve as an early indicator of potential corticospinal tract degeneration, facilitating earlier diagnosis and, eventually, preventative measures. This hypothesis will need to be tested in asymptomatic carriers of highly penetrant, ALS-causing genetic variants. Meanwhile, in affected individuals, beta frequency bursting has potential for use in experimental medicine approaches in ALS, which may lead to use as an outcome measure in therapeutic trials.

## Supplementary Material

fcaf339_Supplementary_Data

## Data Availability

The data that support the findings of this study are available on request from the corresponding author. The data are not publicly available due to containing information that could compromise the privacy of research participants. Example code for data processing is available at: https://github.com/mtrubshaw1/Yoganathan_2025_CMC_ALS_task.
