# Supplementary material for: Reduced beta bursting underpins loss of corticomuscular coherence in amyotrophic lateral sclerosis
Source: Brain Commun. 2025 Sep 9;7(5):fcaf339. doi: 10.1093/braincomms/fcaf339 (PMC12501500; doi:10.1093/braincomms/fcaf339)
Supplement: fcaf339_Supplementary_Data [file fcaf339_supplementary_data.pdf]

Supplementary Material

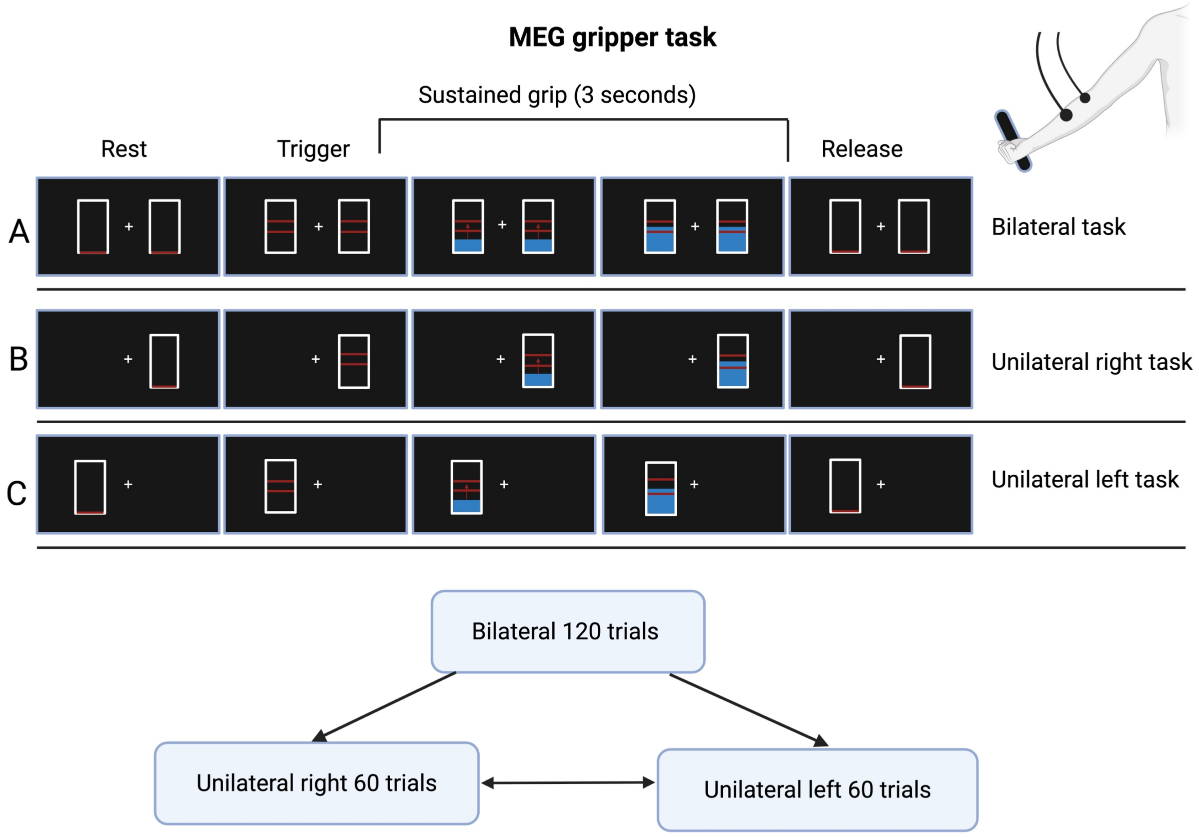

Supplementary Figure 1 – MEG gripper task

Participants (ALS N=42, HC N=33) engaged in three isometric gripper tasks (bilateral, unilateral left hand, and unilateral right tasks), accompanied by concurrent magnetoencephalography (MEG) scanning and bipolar electromyography (EMG) recordings at both forearms, with electrodes placed over the flexor digitorum superficialis muscles. For bilateral gripper tasks, visual cues included two bars on either side of a central fixation cross, indicating the matching force to be exerted by each hand, represented by the height of red lines on each bar. Participants were required to maintain a light grip force for three seconds after the trigger was shown. The grip strength was recorded using MEG-compatible fibre-optic force sensors. For unilateral tasks, a single bar was presented corresponding to the active hand. The effect of handedness was minimised by randomly ordering the right and left task trials throughout the experiment. Participants completed 120 bilateral trials and 60 unilateral trials per side, with a 2000-ms rest interval between trials. Direct visual feedback was provided throughout the task. Created in BioRender https://BioRender.com/exinvy8.

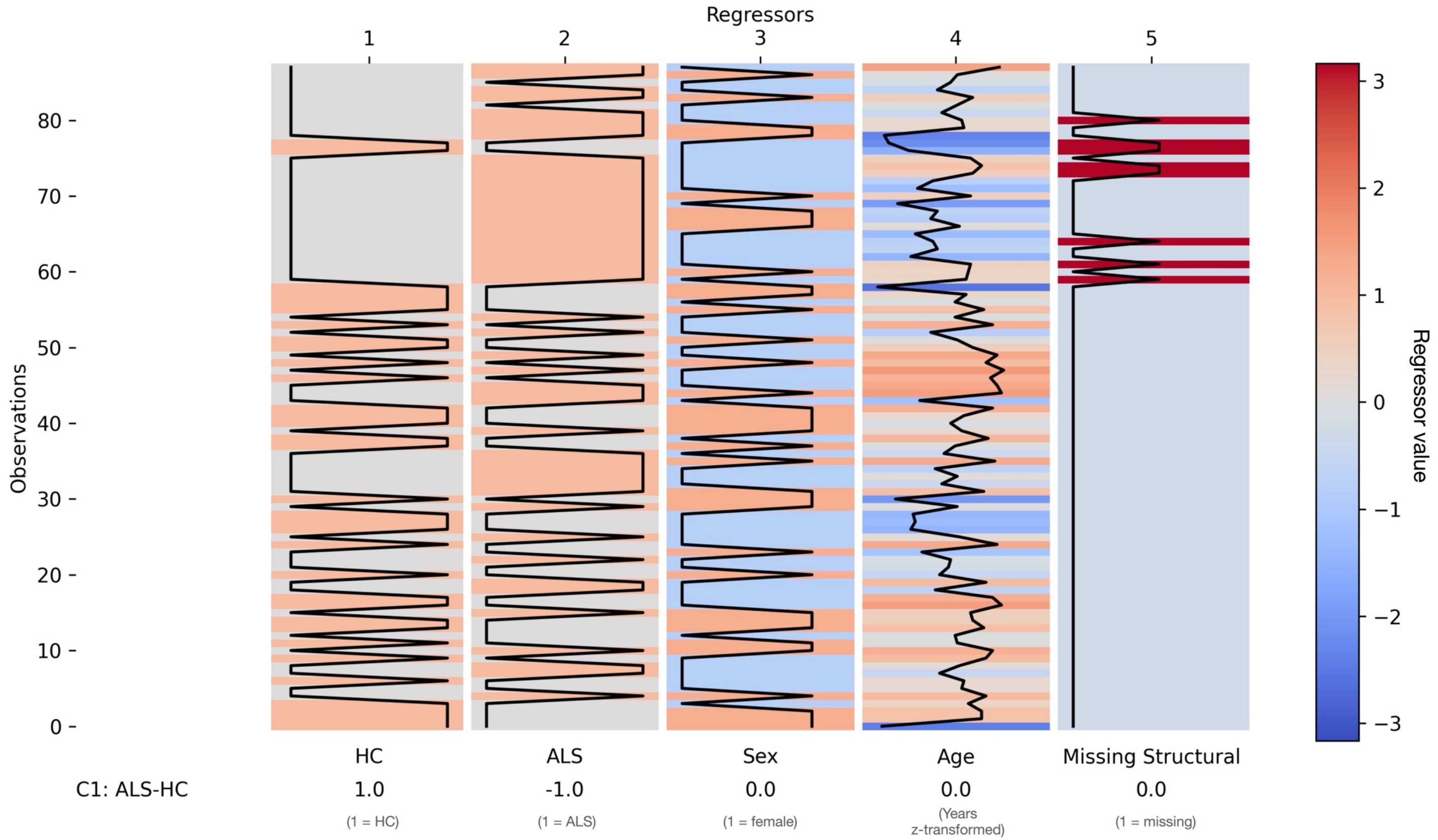

Supplementary Figure 2 – General linear model design for group comparison

This design matrix examined whole-brain beta corticomuscular coherence (CMC) topography and compared beta power and burst metrics between groups. The first regressor models the average beta CMC for healthy controls (HC), while the second regressor captures the average beta CMC for patients with amyotrophic lateral sclerosis (ALS). Additional regressors account for known sources of variability such as age, sex, and the presence of missing structural MRI data among participants. This approach is designed to minimise the influence of these potential confounds on the group comparisons. The confound regressors are standardised by z-transforming the values for age, sex (1=female, 2=male), and missing structural MRI (1=present, 2=absent) across participants.

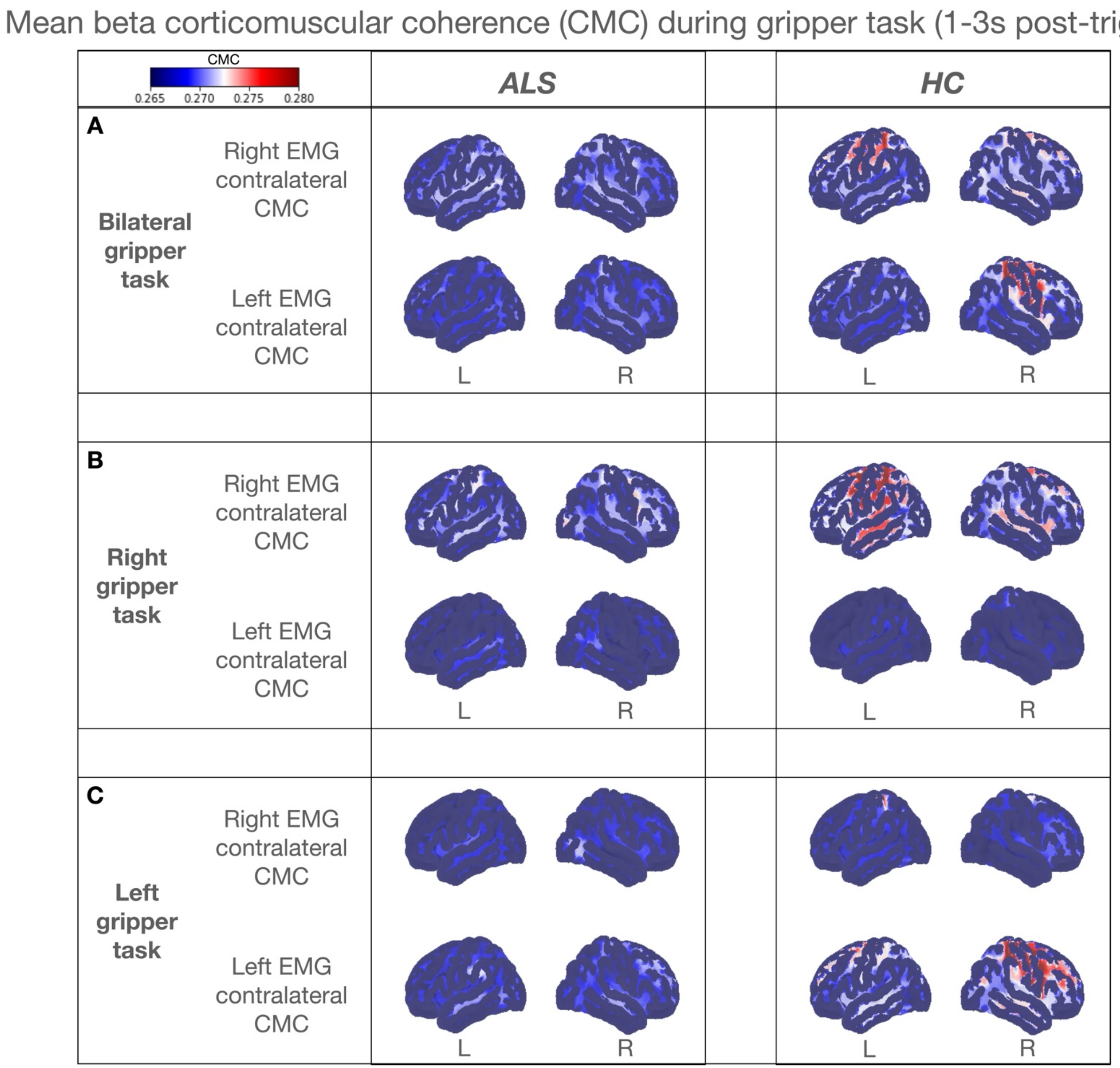

Supplementary Figure 3 – Corticomuscular coherence topography
Topographical maps illustrating the mean contralateral beta corticomuscular coherence (CMC) in ALS (N=42) and HC (N=33) groups during gripper tasks between 1-3 seconds post-grip cue. The maps show coherence in the right and left motor cortices for bilateral (**A**), right (**B**), and left (**C**) gripper tasks. Contralateral CMC is displayed for both right and left electromyography (EMG). Coherence values are color-coded, with a scale from blue to red indicating increasing coherence levels. Regions of clearly high CMC were visible in the HC group in all three tasks, whereas in ALS these did not exist. In response to the bilateral gripper task (**A**), in the HC group, there were regions of high contralateral CMC between the right EMG and left motor areas (**A,** top row), and between the left EMG and right motor areas (**A**, bottom row). In ALS these high-CMC regions were not visible. In response to the right gripper task, in the HC group, there were regions of high contralateral CMC between the right EMG and left motor areas (**B**, top row). In ALS these high-CMC regions were not visible.
In response to the left gripper task, in the HC group, there were regions of high contralateral CMC between the left EMG and right motor areas (**C**, bottom row). In ALS these high-CMC regions were not visible.

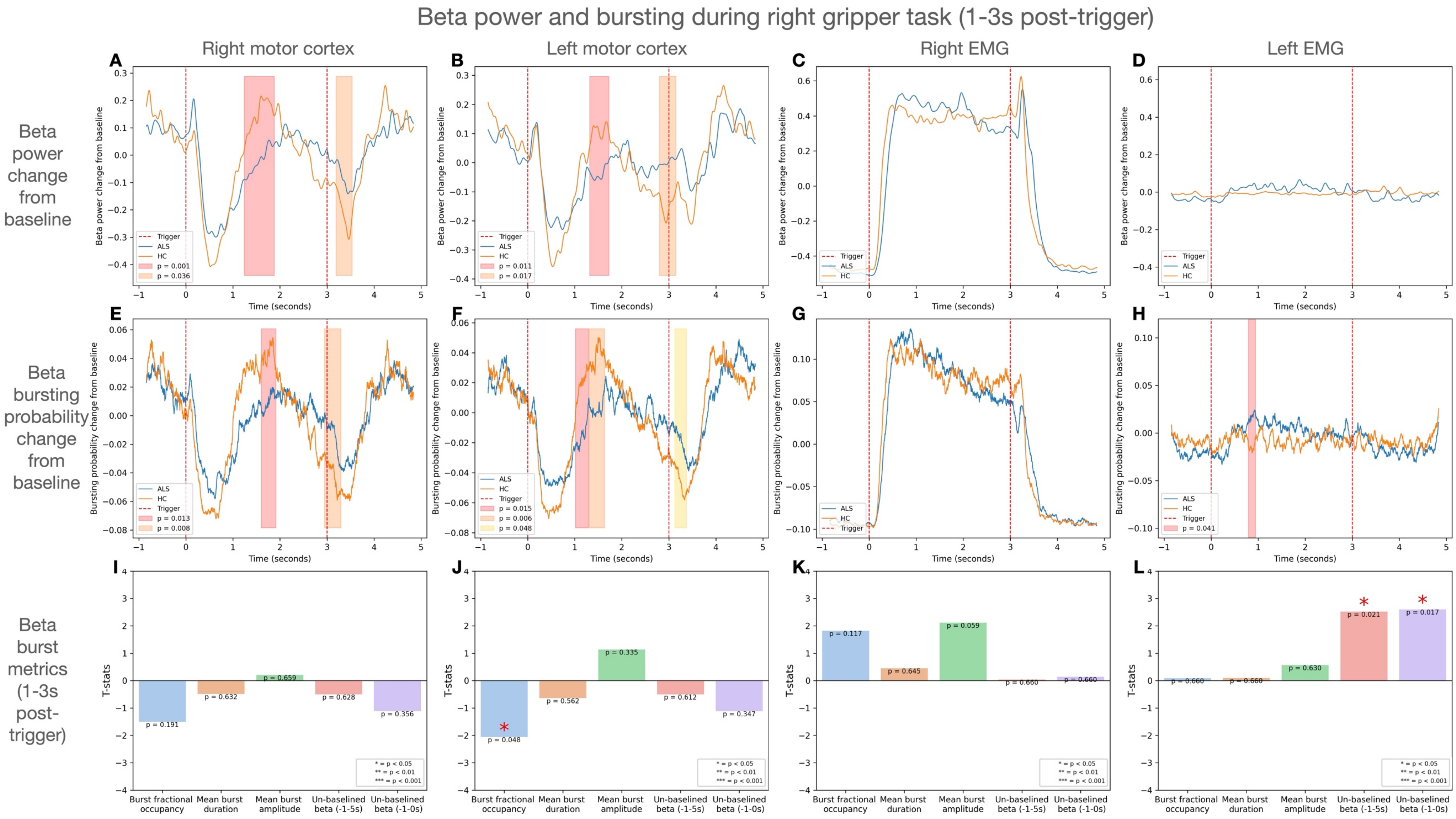

Supplementary Figure 4 - Beta power and bursts in right gripper task

Panels **A, B, C, D** illustrate beta power modulation in the right motor cortex, left motor cortex, right EMG and left EMG respectively with baseline correction across the entire trial period. Time is shown in seconds with the ‘grip’ trigger set at 0s. Shaded vertical bars indicate periods of significant differences from cluster-permutations between groups (ALS N=42, HC N=33) with adjusted p-values marked ($p < 0.05$). Panels **E, F, G, H** display beta bursting probabilities over time in both groups, with baseline correction as previously in the right motor cortex, left motor cortex, right EMG and left EMG respectively. Shaded vertical bars indicate periods of significant differences from cluster-permutations between groups with adjusted p-values marked ($p < 0.05$). Panels **I, J, K, L** display bar graphs comparing beta burst metrics between ALS and HC, between 2-4 seconds. The metrics displayed include burst fractional occupancy, mean burst rate, mean burst amplitude, un-baselined beta power (averaged between -1-5s of the trial) and un-baselined beta power (averaged between -1-0s of the trial). A significant difference ($p < 0.05$) is indicated by an asterisk (*).

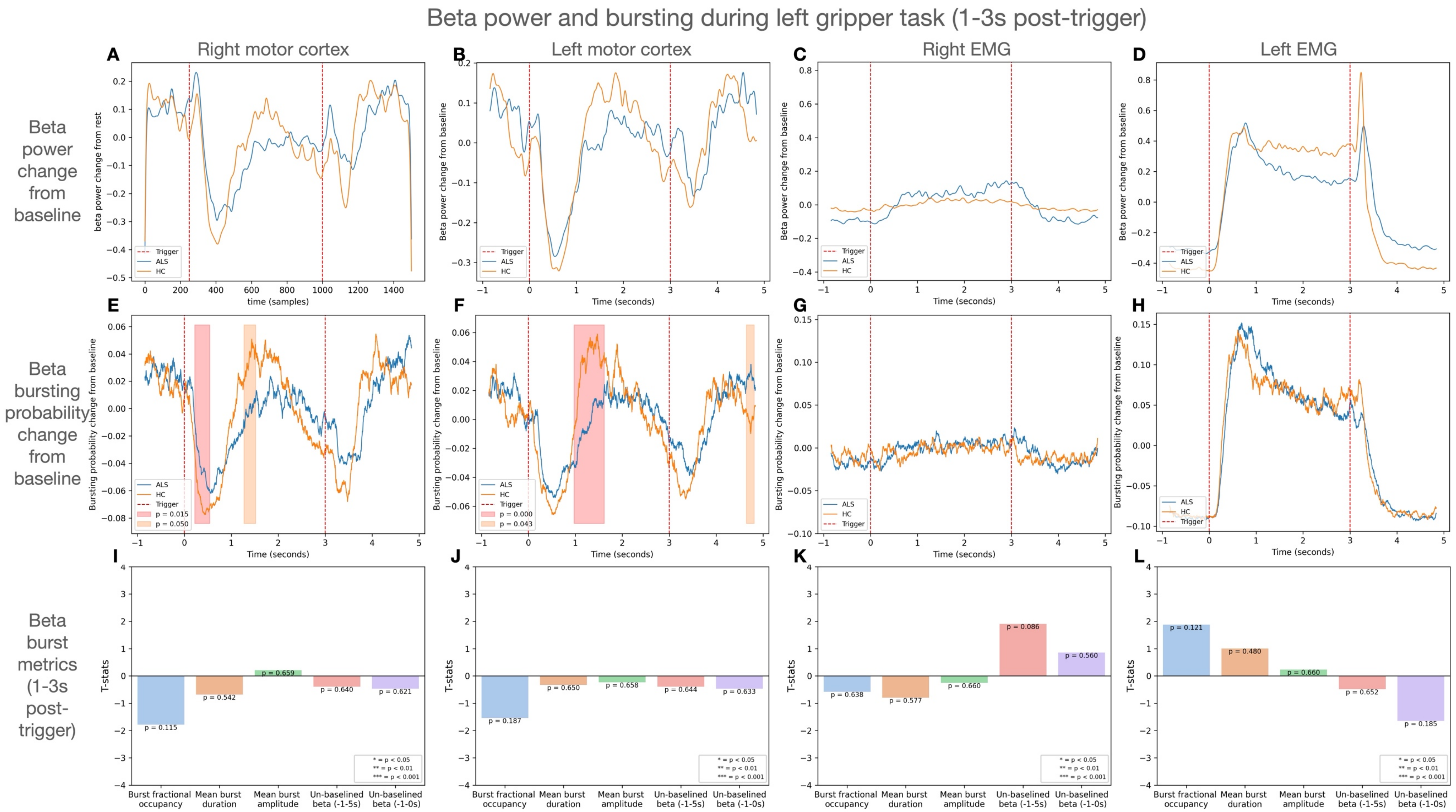

Supplementary Figure 5 - Beta power and bursts in left gripper task

Panels **A, B, C, D** illustrate beta power modulation in the right motor cortex, left motor cortex, right EMG and left EMG respectively with baseline correction across the entire trial period. Time is shown in seconds with the 'grip' trigger set at 0s. Shaded vertical bars indicate periods of significant differences from cluster-permutations between groups (ALS N=42, HC N=33) with adjusted p-values marked ($p < 0.05$). Panels **E, F, G, H** display beta bursting probabilities over time in both groups, with baseline correction as previously in the right motor cortex, left motor cortex, right EMG and left EMG respectively. Shaded vertical bars indicate periods of significant differences between groups from cluster-permutations with adjusted p-values marked ($p < 0.05$). Panels **I, J, K, L** display bar graphs comparing beta burst metrics between ALS and HC, between 2-4 seconds. The metrics displayed include burst fractional occupancy, mean burst rate, mean burst amplitude, un-baselined beta power (averaged between -1-5s of the trial) and un-baselined beta power (averaged between -1-0s of the trial). A significant difference ($p < 0.05$) is indicated by an asterisk (*).

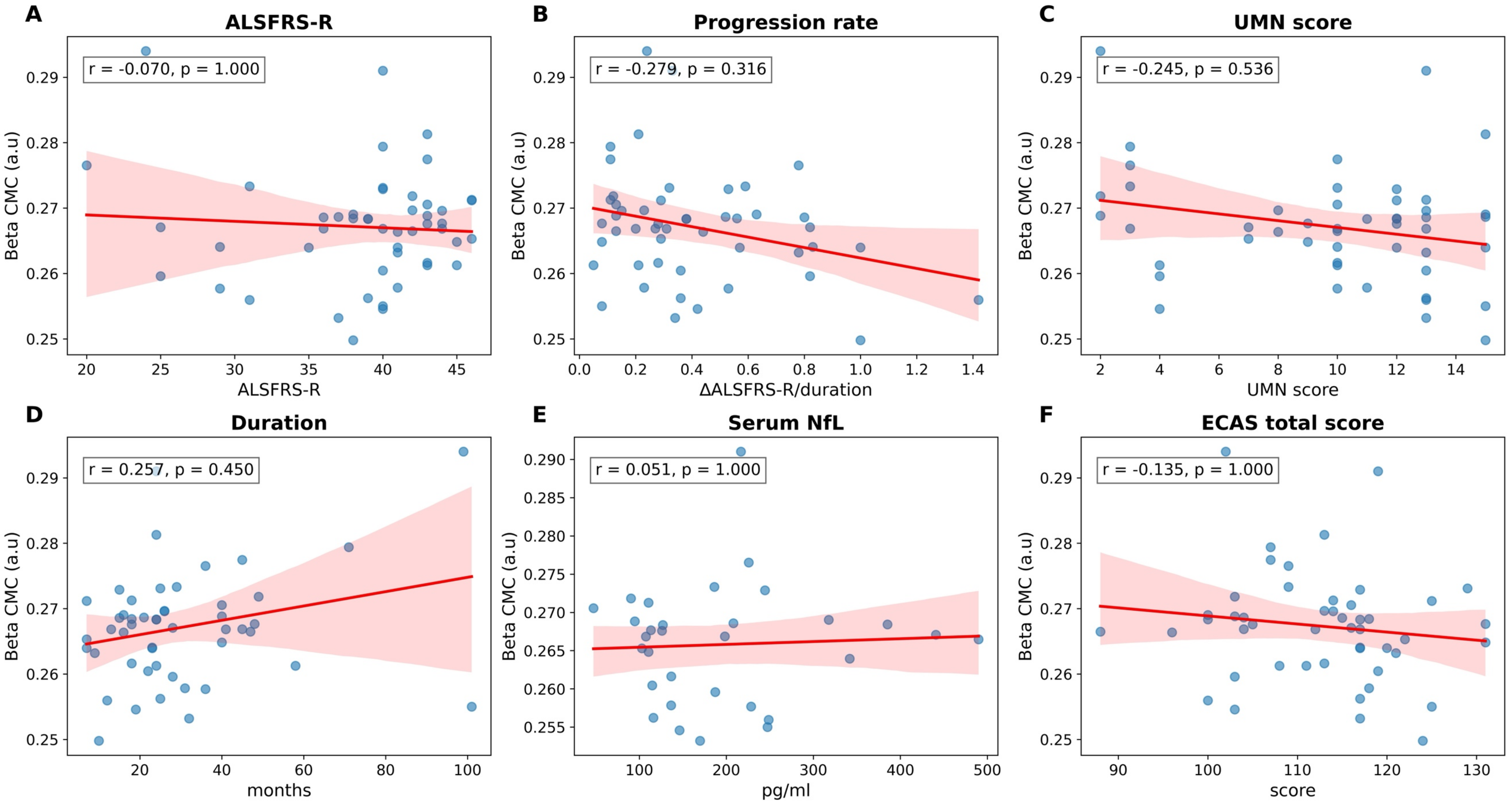

Supplementary Figure 6 - Clinical correlations I

Comparison between beta-band CMC and clinical measures in the ALS group only. Each data point represents an individual ALS participant (N=42). The red regression line represents the best fit line through the data points, calculated using Pearson's r linear regression with p-values adjusted using Bonferroni correction. The shaded area shows the 95% confidence interval. There were no significant correlations between beta-band CMC and ALS measures of disability.

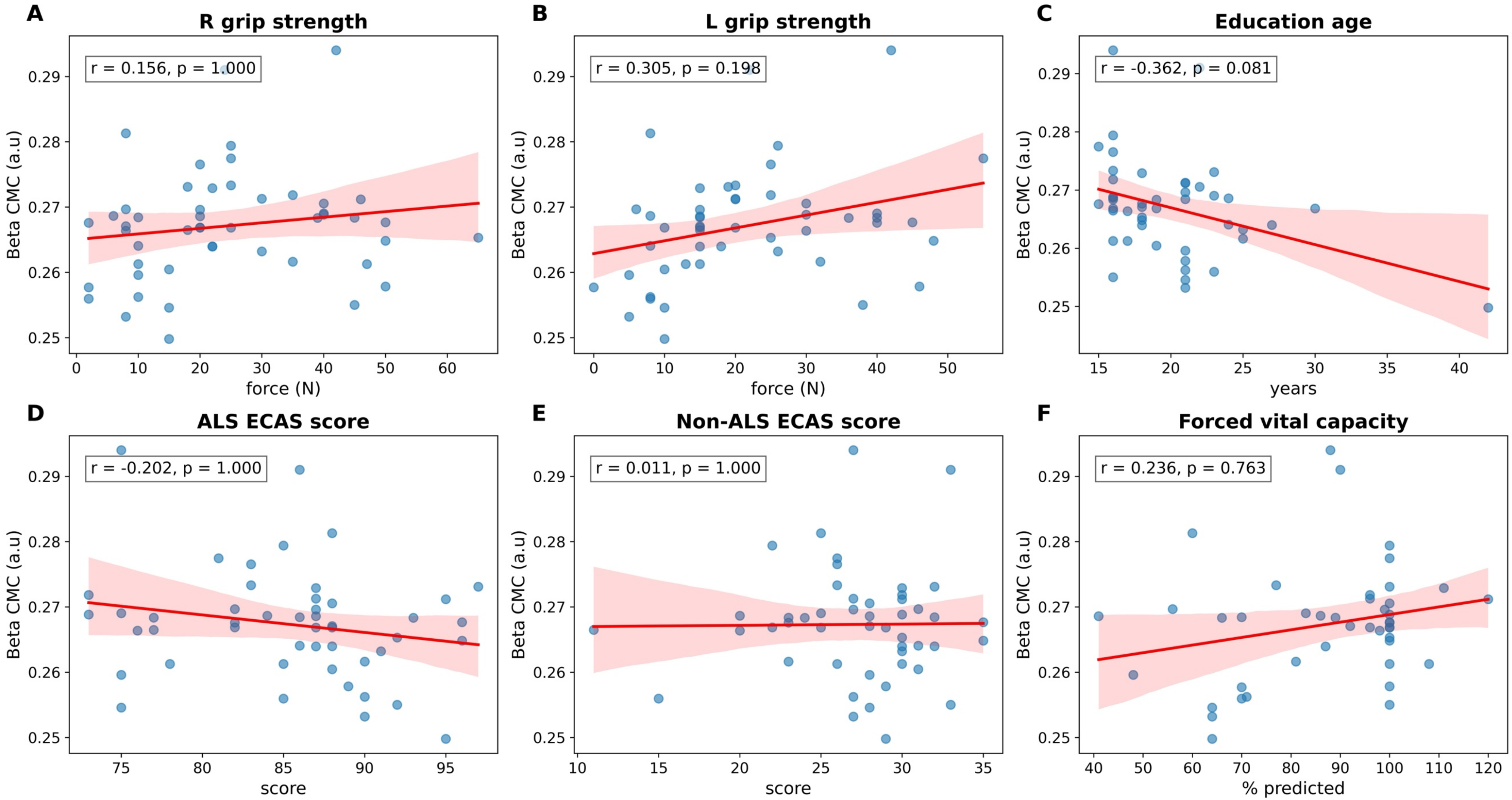

Supplementary Figure 7 – Clinical correlations II

Comparison between beta-band CMC and other clinical measures in the ALS group only. Each data point represents an individual ALS participant (N=42). The red regression line represents the best fit line through the data points, calculated using Pearson's r linear regression with p-values adjusted using Bonferroni correction. The shaded area shows the 95% confidence interval. There were no significant correlations between beta-band CMC and right and left grip strength, years of education, ALS-specific ECAS score, non-ALS specific ECAS score and forced vital capacity.

Mean ± SD	All patients (n=42)	Male ALS patients (n=28)	Female ALS patients (n=14)	Adjusted *p*-value
Onset site of 1st weakness (%)	UL (46%), LL (37%), bulbar (17%)	UL (47%), LL (44%), bulbar (9%)	UL (50%), LL (17%), bulbar (33%)	1.00
Side of weakness (%)	Right (52%), left (35%), both (13%)	Right (59%), left (26%), both (15%)	Right (39%), Left (50%), both (11%)	1.00
Disease duration (months)	30 ± 20	32 ± 22	25 ± 12	1.00
Progression rate (ΔALSFRS-R/duration)	0.4 ± 0.3	0.4 ± 0.3	0.5 ± 0.3	1.00
ALSFRS-R (/48)<br>- Bulbar (/12)<br>- UL (/12)<br>- LL (/12)<br>- Respiratory (/12)	39 ± 6<br>10 ± 3<br>9 ± 3<br>9 ± 3<br>11 ± 2	39 ± 7<br>11 ± 3<br>9 ± 3<br>9 ± 3<br>11 ± 2	38 ± 4<br>9 ± 3<br>8 ± 2<br>9 ± 3<br>11 ± 2	1.00<br>1.00<br>1.00<br>1.00<br>1.00
King's Staging (1-4)	2 ± 1	2 ± 1	3 ± 1	0.11
ECAS score	Normal (81%), borderline (7%), impaired (12%)	Normal (80%), borderline (7%), impaired (13%)	Normal (83%), borderline (6%), impaired (11%)	1.00
UMN Score (/15)	10 ± 4	9 ± 4	10 ± 4	1.00
PEG (yes %)	10%	6%	17%	1.00
NIV (yes %)	6%	7%	6%	1.00
FVC (% predicted)	90% ± 15	93% ± 12	76% ± 23	1.00
Riluzole (yes)	63%	74%	44%	1.00
Genetics	1 *FIG4*, 1 *ATNX2,* 1 *NEK2* and 1 *DCTN1*	1 *FIG4*, 1 ATNX2, 1 *NEK2* and 1 *DCTN1*	*Nil*	-
NfL (pg/ml) (n=36)	194 ± 107	172.2 ± 92	229.2 ± 122	1.00

UL – upper limb, LL – lower limb, ALSFRS-R – ALS functional rating scale – revised; ECAS – Edinburgh cognitive and behavioural ALS screen; UMN – upper motor neuron; PEG – percutaneous endoscopic gastrostomy; NIV – non-invasive ventilation; FVC – forced vital capacity; NfL – Neurofilament light chain.

Supplementary Table 1 - ALS characteristics

This table summarises the clinical characteristics of the ALS study participants, comparing the sex distribution of these characteristics. There are no statistically significant differences between males and females in the ALS cohort in these clinical characteristics, as indicated by the adjusted p-values via Bonferroni correction.
